# Parents' Source of Vaccine Information and Impact on Vaccine Attitudes, Beliefs, and Nonmedical Exemptions

**DOI:** 10.1155/2012/932741

**Published:** 2012-10-02

**Authors:** Abbey M. Jones, Saad B. Omer, Robert A. Bednarczyk, Neal A. Halsey, Lawrence H. Moulton, Daniel A. Salmon

**Affiliations:** ^1^Hubert Department of Global Health, Emory University Rollins School of Public Health, 1518 Clifton Road NE, Atlanta, GA 30322, USA; ^2^Hubert Department of Global Health, Emory University Rollins School of Public Health and Emory Vaccine Center, 1518 Clifton Road NE, Atlanta, GA 30322, USA; ^3^Department of International Health, Institute for Vaccine Safety, Johns Hopkins Bloomberg School of Public Health, Baltimore, MD 21205, USA

## Abstract

In recent years, use of the Internet to obtain vaccine information has increased. Historical data are necessary to evaluate current vaccine information seeking trends in context. Between 2002 and 2003, surveys were mailed to 1,630 parents of fully vaccinated children and 815 parents of children with at least one vaccine exemption; 56.1% responded. Respondents were asked about their vaccine information sources, perceptions of these sources accuracy, and their beliefs about vaccination. Parents who did not view their child's healthcare provider as a reliable vaccine information source were more likely to obtain vaccine information using the Internet. Parents who were younger, more highly educated, and opposed to school immunization requirements were more likely than their counterparts to use the Internet for vaccine information. Compared to parents who did not use the Internet for vaccine information, those who sought vaccine information on the Internet were more likely to have lower perceptions of vaccine safety (adjusted odds ratio (aOR), 1.66; 95% CI, 1.18–2.35), vaccine effectiveness (aOR, 1.83; 95% CI, 1.32–2.53), and disease susceptibility (aOR, 2.08; 95% CI, 1.49–2.90) and were more likely to have a child with a nonmedical exemption (aOR 3.53, 95% CI, 2.61–4.76). These findings provide context to interpret recent vaccine information seeking research.

## 1. Introduction 

High coverage of recommended vaccines in the United States has resulted in a reduction in incidence of greater than 99% for many vaccine-preventable diseases [1–3]. These high immunization coverage levels [4] are due, in large part, to school immunization requirements [5]. While immunization coverage is at or near record highs [6], vaccine refusal rates have increased in states that readily allow nonmedical exemptions to school immunization requirements [7]. Additionally, vaccine refusal is often clustered geographically which has been associated with outbreaks of disease [8]. Increasing exemption rates and the potential for reemergence of vaccine-preventable diseases in the United States highlight the need to effectively communicate accurate information on vaccination to parents. The most common source of vaccine information is primary healthcare providers, but research has shown that parents obtain vaccine information from a multitude of other sources as well [9].

The Internet has rapidly become a widely used source of information, including information on vaccines. While Internet use for information seeking is ubiquitous now, it was a common source of health-related information dating back to the early 2000s. In February 2004, it was estimated that three-quarters of all Americans had access to the Internet [10]. In 2003, the National Cancer Institute estimated that in the prior year, a third of all Americans used the Internet to search for health-related information at least monthly and two-thirds used the Internet for this purpose at least once a year [11]. This widespread use of online information-seeking predated the rise of interactive content and social networking (e.g., Facebook, Twitter) on the Internet, frequently referred to as “Web 2.0” [12, 13]. Web 2.0 has changed the way that people can use the Internet to access health-related information [14]. In this era of Web 2.0, use of the Internet to seek information on vaccines is common [15, 16]. The widespread use of rapidly updated, interactive content has not only increased the potential audience base for Internet based information, it has made it impossible to regulate the information that reaches parents searching for vaccine information. Much of the Internet-based vaccine information that reaches parents contains antivaccine content [17–21], and this is often couched in scientific-sounding language to lend an air of legitimacy to the antivaccine messaging [21].

While it is important to understand which sources are currently used to gather information about vaccines, there is a need for historical context for these findings. For example, we need to understand whether the high level of Internet use regarding vaccine information is a new phenomenon due to the existence of Web 2.0 or if it was common prior to the advent of Web 2.0. Additionally, changes in demographics of those seeking vaccine information over the Internet are important to understand so that future interventions can be appropriately targeted. This study examines the parental attitudes and beliefs associated with Internet use as a source of vaccine information among participants in a survey regarding vaccine exemptions conducted in the pre-Web 2.0 era [22]. 

## 2. Materials and Methods

This study is a secondary analysis of data collected for a previous study to examine refusal of vaccines among children enrolled in elementary schools in four US states. Detailed study procedures and methods are published elsewhere [22]. 

### 2.1. Target Population and Sample Participants

From an earlier study of school nurses on the topic of vaccine exemptions [23], 1,000 schools were sampled across four states (Colorado, Massachusetts, Missouri, and Washington), including the 150 schools with the highest exemption rates per state, 50 schools with the lowest exemption rates per state, and 50 schools randomly selected from the remaining schools in each state. States were selected on their proportion of exemptions compared to other states (high, medium, and low) [23]. Of these 1,000 schools, 112 were selected for a later study of factors related to vaccine exemptions [22], based on having at least five students with exemptions to vaccination. Up to thirteen children with exemptions were randomly selected from each of the 112 schools, resulting in a total of 815 children selected as cases (children with exemptions for 1 or more vaccines). For each case, two fully vaccinated children were randomly selected from the same school and grade to be controls. 

This study was approved by the Institutional Review Board at Johns Hopkins Bloomberg School of Public Health. 

### 2.2. Survey Procedure

Survey packets, including a disclosure letter to indicate consent to participate, were mailed to parents of the selected children by school nurses and school personnel who were trained by the study team. Surveys were returned by mail to the study team at Johns Hopkins. Surveys sent to parents in Massachusetts were mailed in February 2002, and surveys sent to parents in Colorado, Missouri, and Washington were mailed in February 2003.

Surveys mailed to the parents of exempt children contained exemption-specific questions to distinguish surveys completed by parents of children with exemptions from surveys completed by parents of fully vaccinated children; this allowed the researchers to identify students with exemptions without collecting any identifying information. 

### 2.3. Survey Content

Parents of exempt children were asked to confirm that their child was missing at least one vaccine required by their school, the reason for claiming the exemption, and for medical exemptions, they were asked for the medical contraindication. Parents were excluded from data analysis if their child was listed by the school as exempt but they indicated that their child was fully vaccinated or if their child had a valid medical exemption.

Parents were asked to identify which of 16 sources they had used in the past to obtain information about vaccines (healthcare provider's advice; Vaccine Information Statements; professional organizations; alternative healthcare providers; parents and friends; religious leaders and organizations; media; Internet; local or state health departments; US Centers for Disease Control and Prevention; US Food and Drug Administration; vaccine companies; pharmacists; National Vaccine Information Center; Dissatisfied Parents Together; National Academy of Sciences, Institute of Medicine). For each of these 16 vaccine information sources, respondents were also asked, indicate their perceptions of the quality of the source using a 5-point Likert scale (“extremely poor source” to “excellent source”). Parents were free to interpret these responses through their own perceptions of source quality. The survey did not provide specific guidance regarding what was meant by the terms “poor,” “excellent”, and so forth.

For ten diseases with elementary school vaccination requirements (diphtheria, pertussis, tetanus, measles, mumps, rubella, polio, *Haemophilus influenzae* type b, varicella, and hepatitis B), respondents were asked to use 5-point Likert scales to identify their perceptions of (a) the likelihood that an unimmunized child in the United States would get one of these diseases within the next ten years, (b) the severity if an 8-year old were to get one of these diseases, (c) the effectiveness of vaccines in protecting against these diseases, and (d) the safety of the vaccines against these diseases. 

Beliefs about immunization, and trust in healthcare professionals and the government, were measured using series of 14 questions (immunization beliefs), 11 questions (healthcare professionals), and 6 questions (government), assessed using 5-point Likert scales. Beliefs on who benefits from vaccination (the child, the community, doctors, the government, companies that make vaccines) were assessed using individual 5-point Likert scales (“not at all” to a “great deal”).

Categorical demographic data were collected on age, highest level of education completed, gross household income, and race or ethnicity. For analysis, demographic data were dichotomized, using median values for cutpoints (age: 40 years and younger versus 41 years and older; education: some college or less versus college graduate or higher; income: less than $70,000 versus $70,000 and higher; race: white versus all other race/ethnicity categories). Surveys took approximately 30 minutes to complete. 

### 2.4. Data Analysis

The main analysis conducted with data from this survey [22] was a case-control analysis examining factors related to vaccine exemptions. The analysis presented here is an examination of knowledge, attitudes, beliefs, and practices related to vaccination among parents classified by whether they reported using the Internet to seek information on vaccines or not.

For Likert scales assessing the quality of vaccine information sources, immunization beliefs, and who benefits from vaccination, results were dichotomized, using the two highest level responses (e.g., “strongly agree” and “agree”) compared to the other three lower responses.

Questions about disease susceptibility and severity, vaccine protectiveness and safety, and trust in healthcare professionals and government were summarized by calculating the mean score for each category's Likert scale. For disease susceptibility and severity and vaccine protectiveness and safety, one summary score was generated for each category, averaging over all 10 listed antigens and diseases. For questions of trust, one summary score was generated each for healthcare professionals and government, by averaging over the total questions (11 and 6, resp.) for each category. Each of these summary measures was dichotomized by comparing the lowest quartile of scores to all higher scores. 

Logistic regression models were used to compare differences in demographic characteristics, beliefs about the quality of information sources, immunization key beliefs, who benefits from vaccination and perceptions about disease susceptibility and severity, vaccine protectiveness and safety, and trust in healthcare providers and government between respondents who used the Internet as a source of vaccine information and respondents who did not use the Internet as a source of vaccine information. Unadjusted analyses generating odds ratios (OR) and 95% confidence intervals (CI) and adjusted analyses, including the presence or absence of any vaccine exemption as a covariate, generating adjusted odds ratios (aOR) with 95% CI were conducted. Data were analyzed using SAS version 9.2 (SAS Institute Inc., Cary, NC).

## 3. Results

Surveys were returned by 1367 (56.1%) of the 2435 parents of selected children, including 391 (48.6%) of 805 parents of children with exemptions and 976 (59.9%) of 1630 parents of fully vaccinated children. Of the 391 parents of children that were identified by their school as having an exemption to one or more vaccines, 114 were excluded from analysis; 86 indicated that their child was fully vaccinated and 28 respondents provided valid medical contraindications to vaccination. The remaining 277 parents of children with nonmedical exemptions, as well as all 976 parents of fully vaccinated children, were included in the analysis [22]. 

The majority of respondents (*n* = 997, 79.6%) reported using between 2 and 6 sources for information on vaccines, while few reported using only a single information source (*n* = 55, 4.4%). The most commonly used source of vaccine information was the child's healthcare provider (*n* = 1149, 91.7%), followed by Vaccine Information Statements (printed materials from healthcare providers) (*n* = 1052, 84.0%) and parents/friends (*n* = 674, 53.8%). Six vaccine information sources were identified by approximately three-quarters or more of respondents as good or excellent sources of information, including healthcare provider's advice (*n* = 1004, 81.8%) and the US Centers for Disease Control and Prevention and the National Immunization Program (*n* = 911, 81.6%). Among all respondents, 39.9% (*n* = 425) reported that they view the Internet as a good or excellent source of vaccine information. Overall, 249 respondents (19.9%) reported using the Internet as a source of vaccine information (hereafter referred to as “Internet users”; the remaining 1,004 respondents who reported not using the Internet as a source of vaccine information are hereafter referred to as “Non-Internet users”).

Internet users were more likely to have at least a college degree (aOR, 1.49; 95% CI, 1.12–2.00) and have a gross annual household income of $70,000 or higher (aOR, 1.41; 95% CI, 1.04–1.91). There were no significant differences in parental age, race/ethnicity, or choice of child's primary healthcare provider between Internet users and non-Internet users (Table 2). Internet users were more likely to have a child with an exemption to at least one vaccine than non-Internet users (OR, 3.53, 95% CI, 2.61–4.76) (Table 2).

Internet users were significantly more likely than parents who did not use the Internet to believe that the National Vaccine Information Center was a good or excellent source of vaccine information (aOR, 1.69, 95% CI, 1.12–2.55). The National Vaccine Information Center was previously known as Dissatisfied Parents Together, which was also included as an information source of interest in the survey; there was no difference in the perception of Dissatisfied Parents Together across Internet user category. Internet users were also more likely to regard alternative healthcare providers (aOR, 1.55; 95% CI, 1.12–2.14) as a good or excellent source of information. Internet users were less likely than non-Internet users to consider the following good or excellent sources of vaccine information: healthcare providers (aOR, 0.59; 95% CI, 0.42–0.85), Vaccine Information Statements (aOR, 0.49; 95% CI, 0.35–0.69), professional organizations (aOR, 0.56; 95% CI, 0.39–0.80), local or state health departments (aOR, 0.60; 95% CI, 0.43–0.84), and the CDC (aOR, 0.57; 95% CI, 0.39–0.83) (Table 1). 

Internet users more commonly held key beliefs about vaccines that are not supported by scientific research on vaccines than non-Internet users (Table 3). Internet users were less likely than non-Internet users to believe that vaccines strengthen the immune system (aOR, 0.65; 95% CI, 0.46–0.92) and more likely to believe that children get more immunizations than are good for them (aOR, 2.88; 95% CI, 2.03–4.10), healthy children do not need immunizations (aOR, 2.06; 95% CI, 1.28–3.31), immunizations do more harm than good (aOR, 2.47; 95% CI, 1.60–3.81), and that a child's immune system could be weakened by too many immunizations (aOR, 1.74; 95% CI, 1.25–2.43). 

Internet users were also significantly more likely to be opposed to immunization requirements because immunization requirements go against the freedom of choice (aOR, 2.36; 95% CI, 1.64–3.39) and because parents know what is best for their children (aOR, 2.68; 95% CI, 1.75–4.09) than non-Internet users. Additionally, Internet users were less likely to recognize the benefits of immunization for their child (aOR, 0.39; 95% CI, 0.25–0.59) as well as the community benefits from vaccination (for the child's family, playmates, and neighbors) (aOR, 0.53; 95% CI, 0.36–0.76). Relatedly, these parents also had lower perceptions of disease susceptibility, vaccine protectiveness and vaccine safety (Table 4). No significant differences were observed for trust in government or trust in healthcare providers by Internet vaccine information seeking behaviors. 

## 4. Discussion

This analysis provides a historical context for beliefs about vaccine effectiveness and safety, among parents surveyed prior to the widespread utilization of interactive Internet content and social networking (“Web 2.0”). We found that parents who reported using the Internet to obtain information about vaccines were less likely to agree with accepted tenets of vaccine science, less likely to agree that children need or benefit from vaccines, and more likely to have obtained nonmedical exemptions from vaccination for their children. While there have been recent studies about parental attitudes on vaccination [15, 16, 24, 25], it has been difficult to gauge whether these current attitudes have only recently risen to the observed levels or if current findings are highlighting issues that have been more constant over the years and across changing technology platforms, such as the increased use of interactive content.

This analysis indicates a relationship between Internet use and an acceptance of alternative views to traditional medicine about vaccination that predates the rise of Web 2.0 interactive content on the Internet. Approximately 37% of parents who had children with exemptions reported using the Internet for vaccine information in 2002-3, when this survey was conducted. A recent survey of parents in Utah seeking exemptions for their children documented Internet use for vaccine information of only 43% [26].

Among respondents in this survey, parents who used the Internet as a source of vaccine information were more likely to view the advice of an alternative healthcare provider, such as a chiropractor or acupuncturist, as an accurate source of vaccine information. They were also more likely to view the National Vaccine Information Center, a nonprofit organization that questions the safety of vaccination, as an accurate source. Interestingly, there was less support for Dissatisfied Parents Together, the precursor to the National Vaccine Information Center. As Salmon et al. describe, this may be due to the more neutral, less advocacy-oriented name of the National Vaccine Information Center, relative to Dissatisfied Parents Together [22]. Internet users were significantly less likely to view healthcare providers, professional organizations, and governmental health departments as an accurate source of information. This suggests that the Internet was used more often by parents seeking alternative opinions to traditional medicine than by parents who trust their child's physician as a source of information. A 2006 study in Oregon documented higher rates of use of naturopathic providers among vaccine exemptors than non-exemptors, both for parents themselves (49% versus 13%) and for their children (25% versus 2%) [27]. That study was conducted just a few years after the data collection for the analysis presented here, and there is further need to examine these healthcare trust and usage patterns over time. 

An important finding in this analysis is that parents who use the Internet for vaccine information have lower perceptions of disease susceptibility. This reflects a general decrease in the public's concern over vaccine-preventable diseases as they have become less common in the United States [28, 29], but also suggests that a lack of concern over preventable childhood diseases has motivated parents to seek information from additional sources. Parents who used the Internet for vaccine information were also significantly more likely to have low perceptions of vaccine safety and protectiveness. The parents who sought out vaccine information on the Internet in this study were more likely to be higher educated, have a higher household income, and be younger; these demographic patterns fit well with those associated with parents of children who have received none of the routinely recommended childhood vaccines [30]. 

While these data cannot establish causality for the associations, the information provides evidence supporting the potential for improving vaccine information content available on the Internet and other media. Moreover, the association between parental attitudes and use of the Internet as an information source can be bidirectional. The information on the Internet may influence parental attitude and behavior towards vaccines. On the other hand, distrust of vaccination and disapproval of immunization requirements may influence parents to use the Internet as an alternative source of information. The recently released http://www.vaccines.gov/ website provides a forum to promulgate science-based information on vaccines directly to parents and other vaccine consumers. This is one of a diverse set of credible resources on the topic of vaccines, which includes websites such as those from the Vaccine Education Center at Children's Hospital of Philadelphia [31], Parents of Kids with Infectious Diseases (PKIDs) [32], and the National Network for Immunization Information [33] which are good sources of readily accessible information on the safety and effectiveness of vaccines. 

One limitation of this analysis is the potential for nonresponse bias. While there is no way to measure non-response bias in this study, it is likely that parents who felt strongly about immunization were more likely to return the survey than those with neutral opinions. Additionally, the response rate among parents of fully vaccinated children was higher than the response rate among parents of children with exemptions, which could affect the results if factors influencing Internet use differ between these two groups. Another limitation of this study is that information on schools was not collected, to maintain confidentiality. This prevented accounting for similarities of respondents within schools within this analysis. While this would not be likely to influence the overall effect estimates, it could have implications when calculating the precision of these estimates. 

These data were collected before the rise of interactive information sources and social networking sites, and so serve primarily as a benchmark for comparing vaccine attitudes, beliefs and practices in future studies. As information has become easier to find on the Internet, it is likely that parents who use the Internet frequently have encountered vaccine information on the Internet, whether they intended to or not. Therefore, data being collected and analyzed now that simply asks about sources of vaccine information have the potential to miss parents who actively seek out information on the Internet [24, 25]. However, it is important to understand the characteristics of parents who actively seek information on the Internet, as these parents may have a higher likelihood of being influenced by Internet messaging. The data presented here provide information on parental attitudes and beliefs associated with parents who likely sought out vaccine information on the Internet, and provide a baseline on parental Internet use for vaccine information before the expansion of new media, interactive information sources, and social networking sites. 

## 5. Conclusions

The results of this analysis have implications for the role of the Internet in vaccine communication. Because Internet use and nonmedical exemptions are significantly related, it is necessary for the content of Internet-based vaccine information to best target the appropriate audiences and to have the flexibility in presentation formats to meet the needs of all types of consumers.

## Figures and Tables

**Table 1:** Perceptions of vaccine information sources among parents who did or did not use the Internet for vaccine information.

Information source	Internet used as a source of vaccine information		Unadjusted OR (95% CI)	Adjusted OR^†^ (95% CI)
	Yes (*N* = 249)*	No (*N* = 1,004)*		
	Good or excellent source *N* (%)^‡^	Good or excellent source *N* (%)^‡^		
Printed materials from health-care provider (Vaccine Information Statements)	141 (58.0%)	785 (80.9%)	0.33 (0.24–0.44)	0.49 (0.35–0.69)
Professional organizations, such as doctors/nurses' associations	130 (60.5%)	691 (80.0%)	0.38 (0.28–0.53)	0.56 (0.39–0.80)
US Centers for Disease Control and Prevention (CDC) and the National Immunization Program	154 (68.4%)	757 (85.0%)	0.38 (0.27–0.54)	0.57 (0.39–0.83)
Health-care provider's advice	170 (68.8%)	834 (85.1%)	0.39 (0.28–0.53)	0.59 (0.42–0.85)
Local or state health departments	140 (59.6%)	729 (78.0%)	0.42 (0.31–0.56)	0.60 (0.43–0.84)
Religious leaders and organizations	13 (6.5%)	65 (8.0%)	0.79 (0.43–1.47)	0.81 (0.43–1.52)
US Food and Drug Administration (FDA)	105 (50.2%)	526 (61.6%)	0.63 (0.46–0.85)	0.83 (0.60–1.15)
Pharmacists	107 (48.9%)	504 (56.8%)	0.73 (0.54–0.98)	0.97 (0.71–1.33)
Vaccine companies	54 (23.2%)	251 (28.8%)	0.75 (0.53–1.05)	0.98 (0.69–1.40)
Parents/Friends	82 (34.0%)	300 (32.3%)	1.08 (0.80–1.46)	1.03 (0.76–1.41)
Media (TV, radio, newspapers, books, magazines)	77 (32.6%)	273 (29.6%)	1.15 (0.85–1.57)	1.18 (0.86–1.62)
National Academy of Sciences, Institute of Medicine (IOM)	101 (66.0%)	432 (64.5%)	1.07 (0.74–1.55)	1.22 (0.83–1.80)
Dissatisfied Parents Together (DPT)	46 (30.3%)	127 (19.7%)	1.77 (1.19–2.64)	1.22 (0.79–1.87)
Alternative health care providers, such as chiropractors or acupuncturists	104 (50.0%)	271 (33.5%)	1.98 (1.46–2.70)	1.55 (1.12–2.14)
National Vaccine Information Center	152 (78.0%)	600 (76.1%)	1.12 (0.76–1.61)	1.69 (1.12–2.55)
Internet	134 (56.3%)	291 (35.2%)	2.37 (1.77–3.18)	2.45 (1.80–3.32)

*Not all respondents in each Internet use group completed each Likert scale for their perceptions of the listed information sources. *N* and % values presented are relative the non-missing responses for each scale.

^†^Adjusted for presence of any vaccine exemption.

^‡^Counts presented represent the count of non-missing data, and the corresponding percentage is the percent of respondents in each Internet usage group with non-missing data that indicated that a given information source was a “Good or excellent source”. Missing data were not consistent over information source. Odds ratios are calculated based on non-missing results through unadjusted and adjusted logistic regression.

**Table 2:** Demographic characteristics as predictors for Internet use as a source of vaccine information among parents of school aged children.

Characteristics	Internet used as a source of vaccine information		Unadjusted OR (95% CI)	Adjusted OR* (95% CI)
	Yes (*N* = 249)^††^	No (*N* = 1,004)^††^		
	*N* (%)	*N* (%)		
Older parent age^†^	80 (32.7%)	378 (38.3%)	0.78 (0.58–1.05)	0.72 (0.53–0.97)
Higher parent education^‡^	145 (58.2%)	465 (46.3%)	1.62 (1.22–2.14)	1.49 (1.12–2.00)
Higher household income^§^	105 (47.5%)	346 (39.5%)	1.39 (1.03–1.87)	1.41 (1.04–1.91)
Parent race^∣∣^	211 (92.1%)	883 (92.7%)	0.93 (0.54–1.59)	0.78 (0.45–1.37)
Child's primary healthcare provider^¶^	39 (15.9%)	89 (9.0%)	1.91 (1.27–2.86)	1.27 (0.83–1.96)
Child is exempt for one or more vaccines**	105 (42.2%)	172 (17.1%)	3.53 (2.61–4.76)	N/A

*Adjusted for exemption status.

^†^Parent age is above the median (41 years or older) compared to younger.

^‡^Parent's education level is above the median (college graduate or higher) compared to lower education levels.

^§^Total household income is above the median ($70,000 or higher) compared to lower household income.

^∣∣^Parent's race is white compared to all other races/ethnicities.

^¶^Child's primary healthcare provider is not a doctor (nurse, physician's assistant, chiropractor, homeopathic doctor, or other) compared to doctor/physician.

**Child has nonmedical exemption for one or more vaccine compared to fully vaccinated; odds ratios adjusted by exemption status or stratified on exemption status could not be calculated.

^††^Counts presented represent the count of nonmissing data, and the corresponding percentage is the percent of respondents in each Internet usage group with nonmissing data that indicated that a given information source was a “Good or excellent source.” Missing data were not consistent over information source. Odds ratios are calculated based on nonmissing results through unadjusted and adjusted logistic regression.

**Table 3:** Association of vaccination beliefs with Internet use as a source of vaccine information among parents of school aged children.

Key beliefs (agree or strongly agree)	Internet used as a source of vaccine information		Unadjusted OR (95% CI)	Adjusted OR* (95% CI)
	Yes (*N* = 249)	No (*N* = 1,004)		
	*N* (%)^†^	*N* (%)^†^		
Children should only be immunized against serious diseases	152 (62.6%)	577 (60.4%)	1.10 (0.82–1.47)	1.07 (0.79–1.44)
Children get more immunizations than are good for them	139 (59.9%)	246 (27.3%)	3.97 (2.94–5.37)	2.88 (2.03–4.10)
I am concerned that children's immune system could be weakened by too many immunizations	140 (61.1%)	331 (38.7%)	2.50 (1.85–3.37)	1.74 (1.25–2.43)
I am more likely to trust immunizations that have been around for a while	180 (73.5%)	765 (78.6%)	0.75 (0.55–1.04)	1.03 (0.73–1.46)
Immunizations are one of the safest forms of medicine ever developed	61 (26.3%)	350 (39.7%)	0.54 (0.39–0.75)	0.73 (0.52–1.03)
Immunizations are getting better and safer all of the time, as a result of medical research	107 (46.5%)	526 (62.0%)	0.53 (0.40–0.71)	0.75 (0.54–1.03)
Vaccines strengthen the immune system	65 (29.3%)	358 (46.0%)	0.49 (0.35–0.67)	0.65 (0.46–0.92)
It is better for a child to develop immunity by getting sick than to get a vaccine	68 (29.8%)	150 (16.7%)	2.12 (1.52–2.96)	1.31 (0.90–1.91)
Healthy children do not need immunizations	43 (17.6%)	52 (5.8%)	3.75 (2.43–5.77)	2.06 (1.28–3.31)
Immunizations do more harm than good	56 (23.7%)	66 (7.0%)	4.16 (2.82–6.15)	2.47 (1.60–3.81)
I am opposed to immunization requirements because they go against freedom of choice	90 (36.6%)	134 (13.8%)	3.61 (2.63–4.95)	2.36 (1.64–3.39)
I am opposed to immunization requirements because parents know what is best for their children	56 (23.0%)	64 (6.5%)	4.26 (2.88–6.30)	2.68 (1.75–4.09)
Immunization requirements protect children from getting diseases from unimmunized children	114 (48.5%)	690 (74.8%)	0.32 (0.24–0.43)	0.44 (0.32–0.60)
Parents should be allowed to send their children to school even if not vaccinated	141 (58.3%)	286 (30.2%)	3.23 (2.41–4.32)	2.21 (1.59–3.07)

*Who benefits from vaccination (moderate or great deal of benefit) *				
The child	160 (68.4%)	849 (90.2%)	0.23 (0.17–0.33)	0.39 (0.25–0.59)
The community—family, child's playmates, people in the child's neighborhood	152 (65.0%)	792 (84.7%)	0.33 (0.24–0.46)	0.53 (0.36–0.76)
The doctor	120 (56.1%)	460 (56.0%)	1.00 (0.74–1.36)	1.06 (0.78–1.45)
The government	117 (58.8%)	448 (59.6%)	0.97 (0.70–1.33)	0.98 (0.70–1.35)
The companies that make vaccines	219 (92.4%)	807 (89.7%)	1.40 (0.83–2.37)	1.17 (0.68–2.01)

*Adjusted for exemption status.

^†^Counts presented represent the count of non-missing data, and the corresponding percentage is the percent of respondents in each Internet usage group with non-missing data that indicated that a given information source was a “Good or excellent source.” Missing data were not consistent over information source. Odds ratios are calculated based on non-missing results through unadjusted and adjusted logistic regression.

**Table 4:** Association of disease, vaccine, and trust constructs with Internet use as a source of vaccine information among parents of school aged children.

Diseases and vaccines	Internet used as a source of vaccine information		Unadjusted OR (95% CI)	Adjusted OR* (95% CI)
	Yes (*N* = 249)	No (*N* = 1,004)		
	Lowest quartile *N* (%)^¶^	Lowest quartile *N* (%)^¶^		
Disease susceptibility^†^	108 (43.9%)	196 (20.4%)	3.05 (2.27–4.11)	2.08 (1.49–2.90)
Disease severity^‡^	90 (36.7%)	227 (23.1%)	1.93 (1.43–2.61)	1.35 (0.97–1.87)
Vaccine protectiveness^§^	100 (41.0%)	203 (21.2%)	2.58 (1.92–3.48)	1.83 (1.32–2.53)
Vaccine safety^∣∣^	98 (41.0%)	199 (21.2%)	2.58 (1.91–3.48)	1.66 (1.18–2.35)
Trust in healthcare	103 (41.9%)	348 (35.1%)	1.34 (1.00–1.78)	1.25 (0.93–1.68)
Trust in government	73 (29.6%)	236 (24.1%)	1.32 (0.97–1.80)	1.30 (0.94–1.79)

*Adjusted for exemption status.

^†^How likely an unimmunized child in the United States is to acquire vaccine-preventable diseases on a 5-point Likert scale (impossible to very likely)—mean for 10 diseases.

^‡^How serious it would be if an 8-year-old child acquired vaccine-preventable diseases on a 5-point Likert scale (not at all serious to very serious)—mean for 10 diseases.

^§^How protective vaccines are on a 5-point Likert scale (not protective at all to very protective)—mean for 10 vaccines.

^∣∣^How safe children's vaccines are on a 5-point Likert scale (very unsafe to very safe)—mean for 10 vaccines.

^¶^Counts presented represent the count of non-missing data, and the corresponding percentage is the percent of respondents in each Internet usage group with non-missing data that indicated that a given information source was a “Good or excellent source.” Missing data were not consistent over information source. Odds ratios are calculated based on non-missing results through unadjusted and adjusted logistic regression.
